# Beyond Antibiotics: The Role of Antimicrobial Polymers in Modern Therapeutics

**DOI:** 10.3390/pharmaceutics18070821

**Published:** 2026-07-02

**Authors:** Martina Appignani, Giovanni Corfiati, Lisa Marinelli, Marilisa Pia Dimmito, Antonio Di Stefano, Ivana Cacciatore

**Affiliations:** Department of Pharmacy, “G. d’Annunzio” University of Chieti-Pescara, Chieti Scalo, 66100 Chieti, Italy; martina.appignani@phd.unich.it (M.A.); giovanni.corfiati@phd.unich.it (G.C.); marilisa.dimmito@unich.it (M.P.D.); antonio.distefano@unich.it (A.D.S.); ivana.cacciatore@unich.it (I.C.)

**Keywords:** antibiotic, antibiotic resistance, antimicrobial polymer, biofilm

## Abstract

Antimicrobial polymers are a potential and viable alternative to antibiotic therapies, whose effectiveness is increasingly compromised by the onset of antimicrobial resistance and antibiotic persistence. Antimicrobial polymers also have potential in the formulation of new drug delivery systems. Difficulties in finding new naturally derived compounds and classes of antimicrobials pose a major threat to public health and have made finding an alternative crucial. The aim of this review is to provide a comprehensive overview of the antimicrobial polymers currently available, whether of natural or synthetic origin, and to explore their use in pharmaceutical formulations, including a brief description of their preparation methods. Furthermore, a critical assessment of the advantages, limitations, and potential future developments of these systems will be presented, with particular attention to their clinical applicability, safety profiles, and role in combating antimicrobial resistance. Finally, the review highlights key challenges and future directions for the clinical translation of antimicrobial polymer-based systems, with a focus on safety, scalability, and their potential role in addressing antimicrobial resistance.

## 1. Introduction

Given the rise of antimicrobial resistance (AMR) phenomena, there has been a growing interest in the discovery and study of antimicrobial polymers since 1965 [1], when they were synthesized for the first time.

AMR is defined as the ability of pathogenic micro-organisms—such as viruses, bacteria, and fungi—and organisms like parasites to resist antibiotic treatments and consequently survive within host cells, whether animal or human [2]. In terms of doses of effective concentrations, antibiotic resistance occurs when the minimum inhibitory concentration (MIC) is no longer sufficient to determine the mortality of the—up to that point—susceptible pathogenic micro-organism strain. AMR is not the only mechanism of insensitivity to antimicrobial treatments; tolerance mechanisms can also occur. Antimicrobial tolerance consists of a higher minimum duration of killing (MDK) of an antibiotic in a tolerant micro-organism, compared to the MDK for a susceptible micro-organism [3].

Abuse of antibiotics is a documented phenomenon both in those countries where antibiotics can only be purchased on prescription, and in those countries where antibiotics can be purchased without a prescription. The latter increases the phenomenon of taking antibiotics without a prescription, even in countries where the sale of antibiotics is controlled, as it is possible to buy them online. The presence of a prescription requirement alone is not a deterrent for the onset of AMR, as studies have shown that in 30% to 50% of cases, an inappropriate prescription occurs, both in the choice of antimicrobial agent and in the duration of therapy [4]. Another cause of AMR is the use of antibiotics for the treatment of viral infections or conditions such as asthma, where there is no therapeutic indication for the use of antibiotics [5,6]. The overuse of antibiotics in intensive livestock farming and agriculture is another key driver of AMR and represents one of the major threats to public health and global ecological balance. In the United States, it is estimated that approximately 80% of all antibiotics sold are used in animal husbandry, often without a genuine clinical need [7]. These practices are typically justified by the prospect of achieving higher and faster production yields of improved quality. However, the immediate economic benefit is offset by serious long-term health and environmental consequences. The systematic use of antibiotics in agricultural settings promotes the development and selection of resistant bacterial strains, which can be transmitted to humans through the food chain [8,9].

The ability to resist the action of antimicrobials depends on four principal mechanisms: inactivation of antibiotics, modification of antibiotic molecule target, limited uptake, and active efflux of antibiotic molecule. The inactivation of antibiotics can occur in two different ways: enzymatic degradation through production of enzymes which degrade the antibiotic molecule (e.g., production of β-lactamases to degrade β-lactams) [10], and chemical modification of the antibiotic molecule, such as acetylation to make the drug inactive [11]. The modification of the antibiotic molecule’s target involves changes on the bacterial cell itself to escape the antibiotic mechanism of action (e.g., reduction of the affinity of penicillin-binding proteins for β-lactams to counteract their interference with the synthesis of peptidoglycan) [12].

The limited uptake of the antibiotic molecule is performed by decreasing the expression of porins (just like in one of the β-lactam resistance mechanisms), channels that allow the entry of the antibacterial molecule inside the cell, while active efflux of the antibiotic molecule is obtained by overexpression of the efflux pump that extrudes the molecule from the cell [13]. All of these features can be achieved through selected molecular mechanisms of genetic transfer: spontaneous mutations and horizontal gene transfer (HGT). While spontaneous mutations are an inherent characteristic of the DNA replication, HGT can be described as a journey of the DNA, which starts from the outside of the cell, where a strain of naked DNA is released by a donor, and ends with integration or recombination of the strain after the recipient has been selected, starting the transfer of a new genetic trait that enables the future generation of bacteria cells to resist the action of antibiotics [14].

AMR—together with other phenomena that allow pathogenic bacteria to escape from the therapies currently available—is one of the major threats to human health up to the present day, and there are difficulties in counteracting infections caused by pathogenic micro-organisms; these difficulties include finding new naturally derived compounds and new classes of antimicrobials which could outperform antibiotics against which bacteria have developed resistance.

Recent reports indicate that antimicrobial resistance is no longer restricted to conventional bacterial pathogens but increasingly involves multidrug-resistant and extensively drug-resistant strains, significantly limiting therapeutic options and increasing morbidity, mortality, and healthcare costs. The urgent need for alternative therapeutic approaches has stimulated interest in innovative anti-infective strategies, including host-directed therapies, engineered biological systems, and advanced biomaterials designed to circumvent traditional resistance mechanisms [15,16].

Current therapeutic challenges arise from the continuous emergence of new antimicrobial molecules alongside the widespread and growing phenomenon of antimicrobial resistance (AMR), which now extends beyond geographical boundaries [17,18]. For this reason, it is of fundamental importance for modern medicine to devise alternative therapeutic strategies to the use of antibiotics for the treatment of bacterial infections. Alternatives strategies to traditional antibiotic approaches include antimicrobial peptides, bacteriophages, nanoparticles, engineered bacteria, host-directed immunotherapies, and antimicrobial polymers. These innovative platforms aim not only to eradicate pathogens but also to modulate host immune responses and reduce the selective pressure that drives resistance development [19,20,21,22]. Recently, antimicrobial polymers have gained increasing attention as promising tools to address antimicrobial resistance owing to their structural versatility and multifunctional properties, including membrane disruption, targeted delivery, and controlled drug release [23,24]. Although significant progress has been made in their development, a comprehensive assessment of their antimicrobial mechanisms, design strategies, therapeutic applications, and translational potential is still lacking. This review aims to provide a critical and up-to-date overview of antimicrobial polymers, highlighting recent advances, current limitations, and future perspectives for their clinical application. Thus, a classification of antimicrobial polymers based on their origin, followed by structure–function relationships across different classes and their activity against biofilms, persister cells, and multidrug-resistant pathogens, along with translational aspects, safety issues, and emerging technologies are reported. Finally, advanced polymer-based assemblies and their biomedical and environmental applications are also described.

## 2. Antimicrobial Polymers

Antimicrobial polymers were first reported in the literature in 1965 by Cornell R. J. et al., who described the synthesis of homo- and copolymers derived from 2-methacryloxytropolones [1]. The study aimed to evaluate the biological activity of polymers obtained from tropolone-based monomers, a compound already known for its broad biological properties, including antimicrobial effects. The resulting polymers exhibited a broad spectrum of antibacterial activity against several pathogenic strains, including *Staphylococcus aureus*, *Salmonella typhosa*, *Salmonella chloraesius*, *Escherichia coli*, and *Streptococcus pyogenes*, with inhibition zones of 15 mm, 22 mm, 17 mm, 16 mm, and 17 mm, respectively [1].

**Figure 1:**
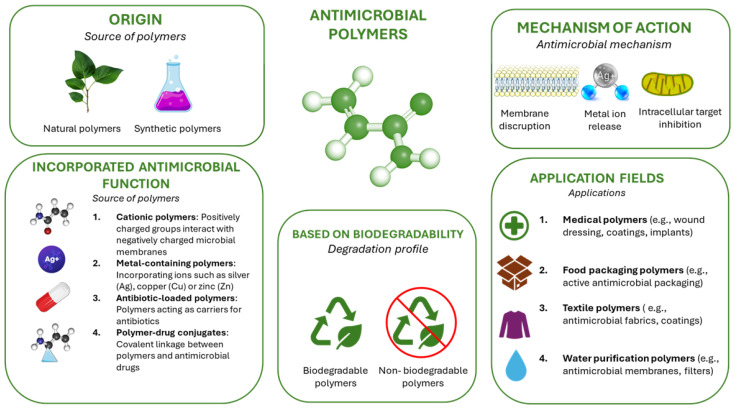
Antimicrobial polymers: **origin**; **mechanism of action**; **functions**; **biodegradability**; and **application fields**. The figure summarizes key aspects of antimicrobial polymers. Origin covers natural, synthetic, and bio-inspired sources. Mechanism of action describes how they inhibit or kill micro-organisms through processes such as membrane disruption or metabolic interference. Functions include antimicrobial activity, antifouling activity, and release of active agents. Biodegradability refers to their degradation pathways and environmental compatibility. Application fields encompass biomedical, environmental, and industrial uses such as coatings, packaging, and drug delivery systems. The figure was generated using Figure Lab software Figure Lab—AI scientific figure platform.

To date, a much broader understanding of antimicrobial polymers has been achieved, allowing their classification according to several criteria (Figure 1, Table 1). These include:

(I) Origin, distinguishing natural and synthetic polymers;
(II) Mechanism of antimicrobial action, such as membrane disruption, metal ion release, or inhibition of intracellular targets;
(III) Type of antimicrobial functionality or incorporated agent, including (1) cationic polymers bearing positively charged groups that interact with negatively charged microbial membranes, (2) metal-containing polymers incorporating ions such as silver (Ag), copper (Cu), or zinc (Zn), (3) antibiotic-loaded polymer systems, and (4) polymer–drug conjugates;
(IV) Biodegradability, distinguishing biodegradable and non-biodegradable systems; and
(V) Application field, including biomedical applications, food packaging, textile engineering, and water purification.

**Table 1:** Classification of antimicrobial polymers according to their origin (natural, synthetic, and hybrid systems) and their principal application domains, including pharmaceutical, food and industrial applications, and emerging biomedical or environmental uses.

Origin	Structure	Pharmaceutical Use	Food/Industrial Use	Other Uses	Ref.
Natural	Chitosan 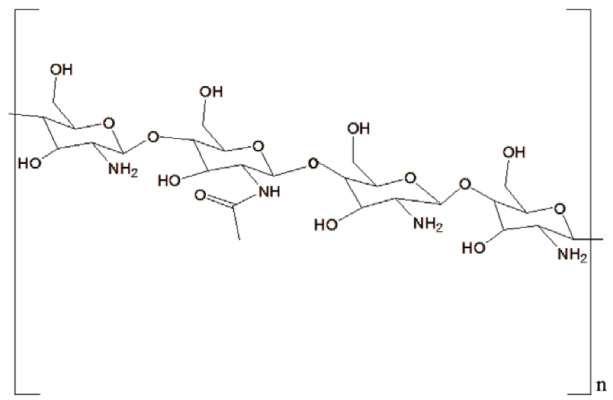	Drug delivery systems in forms like hydrogels, NPs, coated liposomes.	Antibacterial coating for fruits.		[25,26,27,28,29,30,31]
	Epsilon-poly-L-lysine 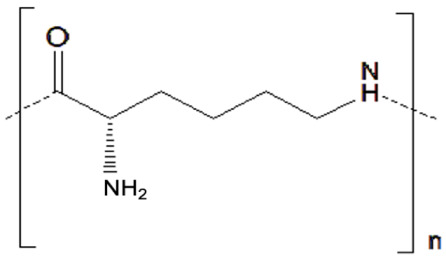	Drug and gene delivery systems, hydrogels.	Food preservative.		[32]
Synthetic	Zwitterionic polymers Phosphorylcholine (PC) ** 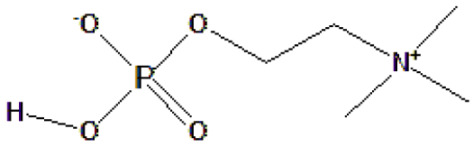 ** Carboxybetaine (CB) ** 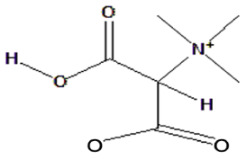 ** Poly(sulfobetaine methacrylate) ** 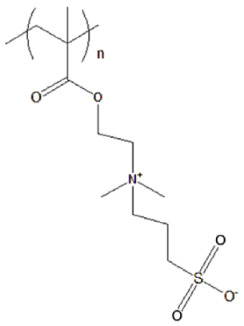 **	Drug delivery systems, surface coatings, wound dressing.	Used in biosensors to detect contaminants.	Water purification system.	[33,34]
	Polymeric ionic liquids (PILs)	Drug delivery system.	Food packaging. Preparation of samples in food analysis.		[35,36,37,38]
	Polyguanidines 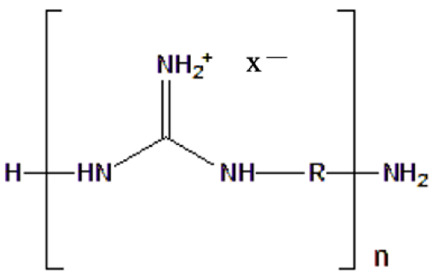	Wound dressing.	Disinfectant in food production.		[39,40]
	Polyethylenimine 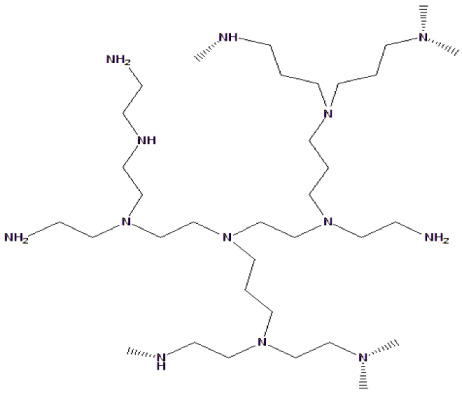	Drug and gene delivery.	Immobilizing agent for enzyme preparation in food processing. Component of food packaging materials.	Water purification system.	[41,42,43]

### 2.1. Natural Polymers

#### 2.1.1. Chitosan

When it comes to natural polymers, it is necessary to mention chitosan, the main chitin derivative (Table 1). Chitosan’s presence in the literature dates to almost two centuries ago: the name “chitosan” was introduced for the first time in 1894 by Hoppe-Seyler, yet it was discovered in 1859 by Charles Rouget, who treated chitin with concentrated caustic potash solution under reflux, obtaining the, at the time, so-called “modified chitin”. This treatment enabled chitin to be soluble in organic acids [44]. Chitosan is the product of deacetylation of chitin, and it is structurally a linear, mucopolysaccharide molecule, composed of *β*-(1→4) glycosidic bonds which link *N*-acetyl-d-glucosamine (Glc*N*Ac) repeated units [45]. It is a biodegradable [46], low-toxicity [47] and biocompatible [48] compound that shows both antimicrobial and fungicidal activity. There are several hypotheses about how chitosan exerts its antimicrobial activity, which is influenced by a variety of factors like pH, molecular weight (MW) and degree of deacetylation (DDA). In terms of the pH of the medium, when pH is lower than pKa, the antimicrobial effect is achieved by electrostatic interaction with the bacterial cell wall thanks to the protonation of amino groups [49]. When pH is higher than pKa, the antimicrobial effect is achieved by hydrophobic interaction and chelation effects, while no significant protonation is observed [50] (Table 2).

**Table 2:** Known targets of natural antimicrobial polymers.

Natural Polymers	Target	Ref.
Chitosan	*Escherichia coli*	[50]
	*Candida albicans*	[51]
	*Staphylococcus aureus*	[51]
	*Candida tropicalis*	[51]
Epsilon-poly-L-lysine	*Lysteria monocitogenes* *Escherichia coli K-12* *Escherichia coli F-2* *Pseudomonas aeruginosa* *Proteus vulgaris* *Aerobacter aerogenes* *Alcaligenes faecalis* *Bacillus cereus* *Bacillus subtilis* *A. simplex* *Corynebacterium xerosis* *Micrococcus lysodeikticus* *Staphylococcus aureus*	[52]

Chitosan’s mechanism of action depends on its MW; in fact, it can penetrate the bacterial cell in accordance with it. If the MW is high, chitosan performs an extracellular antimicrobial effect by chelating essential metals, inhibiting microbial growth [53]; if the MW is low (≤50 kDa), chitosan can penetrate the bacterial cell wall, inhibiting DNA transcription [54]. In the study by Ngo D. H. et al., the antimicrobial activity of chitosan against *S. aureus* and *E. coli* was investigated, and it was found that the antimicrobial activity of chitosan is higher for Gram-negative bacteria when the MW decreases, while for Gram-positive bacteria, it gets higher as the MW increases [55]. Further investigations on other Gram-negative bacteria strains, like *P. aeruginosa* and *P. oleovorans* showed that the low-MW chitosan inhibited the growth of 72.52% compared to the 64.57% of inhibition performed by high-MW chitosan [56]. It has also been demonstrated that low-molecular-weight chitosan can inhibit *Streptococcus mutans* absorption to hydroxyapatite, countering one of the most common bacterial colonizations of tooth surfaces [57].

However, there are plenty of studies that show further possible chitosan mechanisms of action as an antimicrobial compound, and these also depend on the bacterial cell wall structure: chitosan can interact better with more negatively charged bacterial cell walls (Gram-negative bacteria) when pH is below 6.5, whereas there is some evidence that Gram-positive bacteria are more susceptible to chitosan than Gram-negative bacteria (Table 2) [58]. Deacetylation plays an important role in the antibacterial activity of chitosan, since it influences the positive charge density, which determines stronger electrostatic interactions. Edrington T. C. et al. demonstrated a higher DDA occurred alongside higher binding affinity to LPS and higher antimicrobial activity [59].

To assess bacterial or fungal susceptibility to an antimicrobial compound, it is necessary to determine the MIC and MBC (Minimum Inhibitory Concentration and Minimum Bactericidal Concentration). In a study by Yuan Y. et al., the MIC and MBC of three types of chitosan extracted from three different sources were investigated—and compared—against *E. coli* and *S. aureus*. Chitosan obtained by giant river prawn showed the lowest MIC against both bacteria species (0.0625 mg/mL against *E. coli*; 0.0625 mg/mL against *S. aureus*) and the lowest MBC against both bacteria species (0.125 mg/mL against *E. coli*; 0.125 mg/mL against *S. aureus*) [60].

Thanks to its peculiarities, chitosan has a wide range of application fields, from food [61] and agriculture [62] to medicine [63], biotechnology and pharmaceuticals. As regards pharmaceutical applications, chitosan and its derivatives are used as drug delivery systems in a wide variety of forms, including the following:

(1) Hydrogels as a controlled release system for diclofenac sodium to treat inflammation [64];

(2) NPs as a controlled release system for cancer treatment [65], as a gene delivery system [66], and as an antibiofilm system against polymicrobial biofilms of *C. albicans* and *S. aureus* [51];

(3) Coated liposomes as a delivery system of metformin for the treatment of malignant pleural mesothelioma [25].

Chitosan can also be used in the food industry as an antibacterial coating for fruits, since it can protect against numerous pre- and post-harvest diseases of fresh fruits [26].

#### 2.1.2. Epsilon-poly-L-lysine

Epsilon-poly-L-lysine (ε-PL) was first mentioned in the literature in 1977 [67] by Shima S. et al. during the screening of Dragendorff’s reagent when it was found in a culture filtrate of *Streptomyces albulus* (Table 1). ε-PL is a homopolymer composed of 25 to 35 L-lysine residues, and due to the presence of the amino-group at the α-position with strong cationic properties, ε-PL is a strong cationic polymer itself. It has been shown that the number of repeated units of L-lysine correlates with its antimicrobial activity, Shima S. et al. [67] in fact, also performed studies on ε-PL antimicrobial activity, finding out that the MIC (Section 3.2 for MIC values) against a range of bacterial species (Table 2) diminishes with the increase in the number of L-lysine residues. Thanks to this finding, this natural polymer has been employed as a food preservative in Japan since the 1980s. Only a few methods to separate and purify ε-PL from the culture broth have been described:

(1) Separation and purification with the tetraphenylborate anion [68];

(2) Separation and purification through colorimetric determination method of ε-PL using dipicrylamine [69];

(3) Extraction and purification through an alcohol/salt aqueous two-phase system (ATPS) combined with ultrafiltration.

ε-PL exerts its antimicrobial effect through diverse mechanisms of action, such as its interaction with phosphate groups in the inner core of LPS, the principal component of bacterial cell wall. In this proposed mechanism, ε-PL, through electrostatic forces, interacts with LPS of the bacterial cell wall, removing it as if it was a detergent. When ε-PL enters the periplasmic space—it is unknown whether it can interact with the peptidoglycan layer’s components—and reaches a threshold concentration, it promotes the negative curvature of one or both leaflets of the membrane, resulting in the formation of micelles (when only one leaflet curves) and vesicles (when only one leaflet curves) (Table 2) [70]. In a study by Hyldgaard M. et al. where ε-PL has been tested against *E. coli* and *L. innocua*, results showed a stronger antimicrobial effect on *E. coli* both in terms of cell morphology disruption and membrane integrity damage [70]. The MIC for *E. coli* was 74 mg/L, while for *L. innocua* it was significantly higher at 750 mg/L, indicating greater resistance in the latter. Atomic force microscopy (AFM) and transmission electron microscopy (TEM) analysis showed that exposure to ε-PL caused effects such as changes in the stiffness of the bacterial cells and partial removal of the outer cell membrane in *E. coli* cells. Furthermore, ε-PL induces detergent-like disruption of the cell membrane, resulting in the removal of LPS from intact bacterial cells, the extraction of phospholipids from supported lipid bilayers, and the membrane rupture accompanied by the formation of vesicles or micelles. According to these results, a mechanism of action of ε-PL against *E. coli* has been proposed: ε-PL exerts its antimicrobial effect on *E. coli* membranes via a carpet-like mechanism, whereby it interacts with the phospholipid headgroups to induce negative membrane curvature, ultimately leading to the formation of vesicles or micelles. This mechanism is driven by ε-PL’s preferential binding to negatively charged lipid headgroups, which may account for its low cytotoxicity toward mammalian and yeast cells. The observed differences, in this study, in susceptibility among microbial species, suggest that ε-PL efficacy could depend on the membrane lipid composition.

ε-PL has also been tested against *Acinetobacter baumannii*, a bacterium responsible for nosocomial infections, showing that MICs of 1 mg/mL, 50 μL/mL and 20 μL/mL did not inhibit growth. Nevertheless, the 50 μL/mL concentration showed a significant reduction of biofilm formation compared to the antibiofilm effect of polymyxin E and polymyxin B [71]. ε-PL also exerts antifungal activity against a variety of fungi like *Alternaria alternata,* a fungus responsible for postharvest rot (significantly reducing the rot area on apples, tomatoes and jujube fruits) [72] and *C. albicans* (with a MIC of 512 μg/mL, it strongly inhibits the effect of the fungus and biofilm formation, and it blocks the yeast-to-hypha transition) [73].

Given its antimicrobial and antifungal properties, ε-PL has applications in food, medical and biomedical industries: it is used for the improvement of a material’s properties and performance when used as an antimicrobial coating or functionalizing an implant’s surface; it can be part of biocompatible antibacterial and antifungal systems when loaded inside NPs; it is used as a food preservative, being recognized by the Food and Drug Administration as a safe antimicrobial agent [32].

### 2.2. Synthetic Polymers

#### 2.2.1. Zwitterionic Polymers

Zwitterionic polymers are macromolecules whose structure contains both positive and negative charges, which confers on them high polarity (Table 1) [74]. Zwitterionic polymers, such as phosphorylcholine (PC), carboxybetaine (CB), and sulfobetaine (SB) are known to be hydrophilic and biocompatible, characteristics that could make them a valid alternative to polyethylene glycol (PEG), a polymer employed as a drug delivery system and as a coating for several surfaces (Table 3). Recent studies on the use of zwitterionic polymers bonded to NPs have highlighted notable characteristics: SB-siloxane bonded to the silica particle (SiP) surface shows high stability regardless of the temperature and salt concentration [75]. Zwitterionic polymers have the special feature of inhibiting biofilm formation, so they are not usually classified by their MIC. Zwitterionic polymers’ mechanism of action consists in the ability to bind a large amount of water through electrostatic interactions, in order to form a hydrophilic layer which resists bacterial, protein and platelet adhesion, also enhancing the hemocompatibility of the medical devices they are used with [33].

Zwitterionic polymers show a higher long-term performance in repelling bacteria and antibiofouling features compared to other known antibiofouling materials. In a study by Cheng G. et al., it was demonstrated that poly(sulfobetaine methacrylate) (pSBMA) is able to drastically reduce biofilm formation for *Pseudomonas aeruginosa* and *Staphylococcus epidermidis* [76].

Biofouling is the unwanted accumulation and growth of micro-organisms forming biofilms on surfaces [77]. Further studies about zwitterionic polymers’ ability to reduce long-term biofilm formation showed that poly(carboxybetaine methacrylate) (pCBMA) can reduce the biofilm formation of *P. aeruginosa* up to 240 h by 95% at 25 °C and up to 64 h by 93% at 37 °C and suppress *Pseudomonas putida* biofilm accumulation up to 192 h by 93% at 37 °C. Moreover, pCBMA exhibits strong resistance to non-specific protein adsorption from human plasma [78].

Zwitterionic polymers can also be used together with bactericidal agents, such as polymyxin B, to enhance their antimicrobial effects and to prevent protein adhesion on silicone surfaces coated with (pSBMA)/Polymyxin B [33].

The high long-term performance in repelling bacteria and antibiofouling features, together with the ability to reduce biofilm formation, make zwitterionic polymers a perfect candidate to increase the biomedical devices’ performances. Moreover, zwitterionic polymers, thanks to their features, have been extensively used to produce antifouling surfaces for medical devices, biosensors and marine coatings and to modify membrane surfaces to improve the antibiofouling characteristics in the filtration process [34].

**Table 3:** Known targets of synthetic antimicrobial polymers.

Synthetic Polymers	Target	Ref.
Zwitterionic polymers	*Pseudomonas aeuroginosa*	[76]
	*Staphylococcus epidermidis*	
	*Pseudomonas aeuroginosa*	
Polyguanidines	*Bacillus subtilis* *Staphylococcus aureus* *Escherichia coli* *Aerobacter aerogenes* *Pseudomonas aeruginosa*	[79]
Polyethylenimine	*Staphylococcus aureus* (selective)	[80]
	*Escherichia coli*	

#### 2.2.2. Polymeric Ionic Liquids (PILs)

Polymeric ionic liquids, also referred to as PILs, were first mentioned in the literature in 1998 (Table 1) [81]. They are prepared by polymerization of ionic liquid (IL) monomers, and they come in solid form. Some PILs, quaternary ammonium-, pyridinium- and imidazolium-based ionic liquids, show antimicrobial activity against Gram-positive and Gram-negative bacteria, algae and fungi [82]. Three-dimensional printed material can be obtained from PILs, and when coupled with the formation of AgNPs, they show a synergistic antimicrobial greater effect than the bacteriostatic effect of PILs alone [83]. PILs are formed by repeated units of ILs or are composed of ILs and other monomers like styrene, acrylonitrile and divinylbenzene. Given that PILs are basically a chain of repeated ILs, they exert their antimicrobial mechanism like ILs, but a much lower MIC is needed given that PILs can act on different areas of the bacterial membrane. There are two different types of ILs, cationic and anionic. There will only be a description of the cationic ILs, since the anionic ILs are used combined with antibiotics. Cationic ILs exert their bacterial mechanisms by being adsorbed on the bacterial cell wall through its cationic group; this makes the cell wall thinner, destabilizing its structure. In a study by Fang Z. et al., the MIC of imidazolium-, pyrrolidinium-, and pyridine- based ILs and ILs linked to typical antimicrobial agents were compared [84]. The results showed that an increased alkyl chain length decreases the MIC among the same type of ILs. For example, methylpyrrolidinium bromide with an alkyl chain length of 1-alkyl increased from four to 12 showed a decreased MIC from 18.500 mg/L to 6.7 mg/L against *E. coli*.

PILs have gained increasing attention in the pharmaceutical field as a versatile drug delivery system component. The adjustable drug loading, intrinsic biocompatibility, and antimicrobial activity make them effective carriers for therapeutic agents, enabling improved solubility and controlled release. PILs also find applications in bioadhesive materials and biofilm disruptors, as well as functional components in drug synthesis and purification processes [35,36,85]. PILs could also be employed as a component of food packaging, thanks to their antibacterial characteristics that enable them to extend a product’s shelf life [37]; this is not the only application in food industry, because PILs can also be used in solid-phase extraction techniques. Due to their physico-chemical characteristics and their ability to interact with analytes, they are used in the preparation of IL-based adsorbent in food analysis [38].

#### 2.2.3. Polyguanidines

Polyguanidines are a class of antimicrobial polymers described for the first time in 1984 [79] as a new class of polymeric biocides (Table 1). There are several methods to prepare polyguanidines, such as (1) polymerization performed in solution, (2) use of sodium dicyanimide or zinc dicyanimide with diamine salts or water [86], and (3) reaction of diamines and chlorine cyan in organic solvents (i.e., glycerol) to prepare polymeric guanidine hydrochloride [87]. The antimicrobial properties of polyguanidines were found when it was first described that acrylate monomers with pendant biguanide groups showed antimicrobial behavior due to the capacity to interact electrostatically with bacterial cell membranes. The antibacterial activity was higher against Gram-positive bacteria than Gram-negative because of the less complex cell wall structure, which allows polymers with high MW to penetrate [79]. Polyguanidines exert their mechanism of action by being absorbed (thanks to their cationic nature) on the negatively charged bacterial cell membrane, causing damage that leads to the leakage of the inner components (Table 3) [88].

Different types of polyguanidines have been tested on different bacteria to determine their MIC. Polyhexamethylene guanidine hydrochloride (PHGC), polyhexamethylene biguanidine hydrochloride (PHBGC), polyhexamethylene guanidine stearate (PHGS), and polyhexamethylene biguanidine stearate (PHBGS) have been tested against ten species of bacteria. Only three will be listed here: they showed, respectively, MICs of 1.55 μg/mL^−1^, 0.78 μg/mL^−1^, 6.25 μg/mL^−1^ and 50 μg/mL^−1^ against *B. subtillis*; MICs of <0.39 μg/mL^−1^, <0.39 μg/mL^−1^, 12.5 μg/mL^−1^ and 0.78 μg/mL^−1^ against *S. aureus*; and MICs of 2.5 μg/mL^−1^, 25 μg/mL^−1^, 100 μg/mL^−1^ and 200 μg/mL^−1^ against *E. coli* [89]. The antibacterial features of polyguanidines could lead to their application in the preparation of novel wound dressings like hydrogels [39], and as a component of disinfectants against fungi in food production: it has been found that polyhexamethylene guanidine hydrochloride has both fungicidal and bactericidal properties against *Enterobacter cloacae* and *Aspergillus tamarii*, micro-organisms which contaminates raw ingredients like cocoa beans. This could make it a good candidate to develop a disinfectant for cocoa beans after harvesting [40].

#### 2.2.4. Polyethylenimine

Polyethylenimine (PEI) is a polymer formed by a branched or linear structure composed of repeating units of ethyleneimine monomers (Table 1). PEI contains primary, secondary, and tertiary amine groups, which contribute to its high cationic charge density. This structure enables PEI to interact with negatively charged surfaces and molecules. PEI can be synthesized in linear form through ring-opening polymerization of 2-ethyl-2-oxazoline followed by hydrolysis [90]; the branched form of PEI can be synthesized through acid-catalyzed polymerization of aziridine [91]. PEI shows antimicrobial activity, and furthermore, it seems to be selective against *S. aureus* [80]. Some results have shown that membrane rupture is not the unique mechanism through which PEI exerts its antimicrobial activity; however, membrane rupture remains one of the most widely accepted hypotheses. *S. aureus* and *S. epidermis* are susceptible to the action of PEI with a MIC of 0.763–12,500 mg/L [42,92]. Thanks to its cationic nature, PEI promotes electrostatic interactions with negatively charged viruses, which cause their removal; for this reason, it can be considered a promising surface modification agent since it has been demonstrated that it improves the antiviral performance of commercial polyether sulfone microfiltration membranes [43]. Moreover, it can be used as an immobilizing agent for enzyme preparation in food processing and as a component of a smart packaging which, when stimulated by a given pH, releases PEI to control bacterial growth to preserve food (Table 3) [41].

### 2.3. Antimicrobial Polymers: From Molecular Design to Biological and Clinical Performance

Despite the wide diversity of antimicrobial polymers, their mechanisms of action largely converge toward membrane disruption or anti-adhesive surface shielding, although their efficiency and translational potential vary significantly (Table 4). Natural polymers such as chitosan and ε-poly-L-lysine act mainly through electrostatic interactions and offer high biodegradability and biocompatibility. However, their antimicrobial performance is strongly influenced by environmental conditions such as pH and ionic strength, which may limit reproducibility in clinical settings [93].

In contrast, synthetic polymers, including polyguanidines, polyethylenimine, and polymeric ionic liquids, generally exhibit higher antimicrobial potency and tunable physicochemical properties. Nevertheless, concerns related to cytotoxicity, long-term environmental persistence, and regulatory approval remain key barriers to clinical translation [94]. Zwitterionic polymers represent a distinct class characterized by anti-adhesive and antifouling behavior rather than direct bactericidal activity, making them particularly suitable for long-term biomedical devices but less effective for acute infection control.

Structure–function relationships are strongly influenced by molecular architecture. Key parameters such as charge density, molecular weight, hydrophobicity, topology, and degradability critically determine biological activity. Highly cationic polymers (e.g., polyguanidines, PEI, and ε-poly-L-lysine) show strong interactions with negatively charged bacterial membranes, leading to membrane destabilization and intracellular leakage. However, increased charge density is often associated with higher cytotoxicity, highlighting the need for an optimal balance between efficacy and safety. Branched architectures generally enhance local charge density and antimicrobial activity compared to linear analogues, while crosslinked systems tend to favor surface-based anti-adhesive effects.

Degradability is another key factor for translational applications [95]. Biodegradable polymers such as chitosan and ε-poly-L-lysine offer improved safety and reduced environmental impact but may suffer from limited long-term stability. In contrast, non-biodegradable synthetic systems provide prolonged activity but raise concerns regarding accumulation and toxicity.

Biofilm-associated infections remain a major challenge due to the protective extracellular polymeric substance (EPS) matrix, which limits diffusion and creates heterogeneous microenvironments that promote persister cell formation. Consequently, MIC/MBC values obtained in planktonic systems do not fully reflect real-world performance. Polymer–biofilm interactions are governed by electrostatic and hydrophobic forces, but EPS components such as extracellular DNA and polysaccharides can significantly reduce penetration efficiency [96]. Natural cationic polymers like chitosan and ε-poly-L-lysine mainly act on biofilm surfaces through electrostatic interactions but show limited penetration into mature biofilms, often resulting in partial biomass reduction rather than complete eradication. Similarly, synthetic cationic polymers exhibit stronger disruption of biofilm structure but may be sequestered by EPS components, limiting deep diffusion. Zwitterionic polymers instead prevent biofilm formation through hydration layer formation but do not disrupt established biofilms.

A major limitation of most antimicrobial polymers is their reduced efficacy against persister cells, which exhibit metabolic dormancy and low membrane activity [97]. Since polymer activity often relies on membrane disruption, these dormant subpopulations can survive treatment and drive infection relapses.

Although multidrug-resistant (MDR) bacteria are not inherently less susceptible to cationic polymers, their enhanced biofilm formation, altered membrane composition, and increased EPS production can reduce polymer accessibility and binding efficiency. This contributes to the discrepancy between strong planktonic activity and limited biofilm efficacy.

Despite promising in vitro and preclinical results, clinical translation remains limited, with most applications restricted to medical devices, wound dressings, and drug delivery systems [98]. Robust clinical trials targeting biofilm-associated infections are still scarce, and regulatory classification remains complex due to the hybrid nature of antimicrobial polymers as materials, devices, and active agents.

Manufacturing challenges further hinder translation, particularly variability in natural polymers and strict synthesis requirements for synthetic systems. Sterilization processes may also alter polymer structure and performance. In addition, the lack of standardized biofilm models limits comparability across studies.

Finally, although natural polymers are generally cost-effective and scalable, synthetic systems often provide superior antimicrobial performance at higher production costs. Overall, successful clinical translation requires balancing antimicrobial efficacy, cytotoxicity, scalability, regulatory compliance, and economic feasibility.

**Table 4:** Comparative quantitative and functional overview of antimicrobial polymers.

Polymer Class	Representative Polymer	Mechanism of Action	MIC (Typical Range)	Biofilm Activity	Cytotoxicity	Selectivity Index (SI)	Degradability	Translational Relevance
Natural cationic polymer	Chitosan	Electrostatic interaction, membrane permeabilization, metal chelation	0.0625–2 mg/mL (species- and MW-dependent)	Moderate–high inhibition	Low	High (generally favorable)	High (biodegradable)	Limited by pH dependence and variability
Natural polycationic peptide	ε-Poly-L-lysine	Detergent-like membrane disruption, LPS interaction, carpet mechanism	~0.074–1 mg/mL (variable by strain)	High inhibition of biofilm formation	Low–moderate	Moderate–high	Biodegradable	Strong potential in food and coatings
Synthetic polycation	Polyethylenimine (PEI)	Membrane disruption, electrostatic binding to anionic membranes	0.7–12,500 mg/L (structure-dependent)	Moderate antibiofilm activity	High (especially branched PEI)	Low–moderate	Non-biodegradable	Limited by toxicity despite efficacy
Synthetic polycation	Polyguanidines (e.g., PHMB, PHGC)	Membrane disruption, leakage of intracellular contents	<0.39–50 µg/mL	High antibiofilm and biocidal activity	Moderate	Moderate	Low	High topical/industrial relevance
Polymeric ionic liquids (PILs)	Imidazolium/pyridinium-based PILs	Membrane destabilization via cationic surface adsorption	~mg/L–low mg/mL (variable)	High (strain-dependent)	Moderate–high (structure-dependent)	Moderate	Low	Promising but toxicity and regulation limit use
Zwitterionic polymers	CB, SB, PC-based polymers	Anti-adhesion via hydration layer formation (non-bactericidal)	Not applicable (non-MIC based)	Very high antibiofouling (up to 90–95% reduction)	Very low	High (indirect effect)	Variable (often low degradation)	Excellent for long-term devices, not killing agents

## 3. Natural and Synthetic Antimicrobial Polymer-Based Assemblies

### 3.1. Hydrogels

Hydrogels are a mesh network composed of a large amount of water, often cross-linked to polymer networks; to date, they represent an alternative to conventional drug formulations as they function as a particular drug delivery system that enables them to easily encapsulate hydrophilic drugs. The direct consequences of the water-absorbing capacity are flexibility and softness [52,92,99]. Hydrogels can be of different sizes and have different architectures, and they can be composed of natural, synthetic or semi-synthetic polymers. Hydrogels are formed by cross-linked polymer chains, such as cellulose, alginate, collagen, fibrin, gelatin and many others [100], together with antimicrobial polymers. Antimicrobial polymers find their application in the formulation of hydrogels as they can represent an additional advantage. Chitosan, for example, although it is used together with other components because of its reduced mechanical strength, is used in the formulation of bioink—for example, ethylenediaminetetraacetic acid (EDTA)—before the addition of Ca^2+^ to increase the amount of chitosan-Ca^2+^ crosslinking [101,102]. In the literature, the antimicrobial nature of chitosan is not the focus of attention when it comes to its use in hydrogel formulations. Instead, ε-PL is used in the patented formulation of hydrogels with antimicrobial, disinfectant and antiviral properties [103].

### 3.2. Nanoparticles

The discovery of nanoparticle (NP) drug delivery systems precedes the very conceptualization of what we know today as “nanotechnology”, introduced and theorized by Nobel prize recipient Richard Feynman. The first references to colloidal transport systems were made in 1961 and subsequently, in 1978, in studies by R.C. Oppenheim and in the early 90s, when a method to produce lipid solid microspheres with a narrow size distribution was patented. Both past and recent studies have a common goal: to create NP drug delivery systems that are chemically stable, allow the solubility and subsequent encapsulation of the cargo, allow a controlled release of the cargo, are biocompatible and can reach the site of interest [104,105,106].

NPs can be distinguished into seven classes: (1) liposomes [107], made of a lipid bilayer membrane surrounding a watery interior (which can be divided into three categories themselves, which are oligolamellar liposomes, large unilamellar liposomes and multilamellar liposomes); (2) emulsions [108], oil-in-water type mixtures; (3) ceramic NPs [109] such as silica and titania; (4) metallic particles [110] like iron oxide NPs; (5) gold-shell NPs [111], a dielectric core covered by a thin gold shell; (6) quantum dots [112], NPs made of semiconductor materials with fluorescent features; and (7) polymeric and lipidic NPs [27,28]. This classification can be simplified by categorizing NPs according to the materials they are composed of; in this case, they would be categorized into inorganic, lipidic or polymeric NPs. Polymeric NPs have several advantages, such as the ability to encapsulate the cargo in their core, the ability to encapsulate cargos of different molecular weights and the ability to function as excellent co-delivery systems [48,109]. NPs made of antimicrobial polymers therefore encompass all of these characteristics, together with the advantage of the potential antimicrobial action, which would make them a valid alternative to available therapies for the treatment of infectious diseases.

A perfect example of antimicrobial NPs is the chitosan-based NPs. Chitosan-based NPs have different applications, such as in wound dressings [29] and tissue engineering, thanks to their ability to promote wound healing, to reduce inflammation and to provide a protective effect by forming a barrier against infection [30].

Another class of polymer-based NPs is cationic NPs like PEI NPs. As described above, PEI has the capacity to disrupt bacterial cell walls. The advantage of PEI NPs is that they can disrupt biofilms [113] and so outperform traditional antibiotic treatments given that biofilm shows resistance to antibiotics [114].

Chitosan NP antimicrobial activity has been investigated against *E. coli* O157 and *L. monocytogenes*. Two different types of NPs—SA1 and SA2—have been tested, because they were made of chitosan obtained from different sources. The differences between these two types of chitosan lie in the MW (SA1 had a MW of 50–190 KDa and SA2 had a MW of 90–190 KDa) and in the DD (SA1 had a DD of 75–85%, SA2 had a DD ≥ 75%). SA1 NPs showed a MIC of 1.2 mg/mL against *E. coli* and 0.04 mg/mL against *L. monocytogenes*, and these results were also highly replicable, unlike the results shown by SA2, which were not replicable [31].

NPs made of ε-PL have also been tested against *S. aureus* and *C. albicans*, showing a MIC of 400 μg/mL against both species [51].

#### PEG-PLGA

PEG-PLGA is the abbreviation for polyethylene glycol-poly lactic acid-co-glycolic acid, a copolymer that has the characteristics of PEG and PLGA [115]. The former endows stability and hydrophilic properties, whereas the latter provides biodegradability and hydrophobic properties. PLGA serves as the biodegradable matrix where hydrophobic and hydrophilic molecules will be encapsulated in the bioderived polymer to be released through hydrolysis. This process degrades the polymer into lactic acid and glycolic acid, which the body metabolizes.

Over the past few years, the global landscape for pharmaceutical applications that rely on PEG-PLGA [116] has greatly expanded, contributing to its ascension as the golden standard for controlled release system design (Table 5). To enable high bioavailability of antimicrobial agents and improve the selective delivery of cargo to infection sites, PEG-PLGA is primarily incorporated into nanoparticles, micelles and hydrogels. The use of PEG-PLGA for pharmaceutical formulations has been shown to significantly improve antimicrobial activity through a reduction in MIC and a favorable modulation of pharmacological release. Several studies have shown that PEG-PLGA acts as a vector to improve the effectiveness of antimicrobial drugs. In particular, the work of Ramôa A.M. et al. suggests that the encapsulation in PEG-PLGA of vancomycin and ciprofloxacin significantly reduces the MIC against strains of *S. aureus* or *P. aeruginosa*, compared to non-encapsulated drugs [117]. The system maintains peptide-like free activity and achieves an MIC of 8–16 μg/mL against *P. aeruginosa* and 16–32 μg/mL against *S. aureus*. The killing capacity was also increased from 12 h to 15 min for *P. aeruginosa* and from 6–8 h to 0.5–1 h for *S. aureus* [117]. Therefore, the mechanism of action of PEG-PLGA is not based on direct antimicrobial activity but is mainly vehicular and protective. There is an improvement in the intracellular distribution of the drug and a facilitation of its accumulation at the infected site [118]. In addition, the use of this system, allowing more selective action against pathogenic bacteria, helps to reduce therapeutic doses and limits the emergence of resistance.

**Table 5:** Methods to prepare PEG-PLGA-based formulations.

Method	Description	Advantages	Disadvantages	Ref.
Single Emulsion (O/W)	A hydrophobic drug and PLGA are dissolved in an organic solvent, then emulsified in an aqueous phase containing a stabilizer, followed by solvent evaporation to form nanoparticles.	- Suitable for encapsulating hydrophobic drugs - Simple and reproducible	- Potential residual solvent toxicity - Limited encapsulation efficiency for hydrophilic drugs	[119]
Double Emulsion (W/O/W)	Hydrophilic drugs are dissolved in an aqueous solution, emulsified into an organic phase containing PLGA, then re-emulsified in an aqueous phase, followed by solvent evaporation.	- Effective for encapsulating hydrophilic drugs - Enhanced drug loading	- Complex process - Possible low encapsulation efficiency - Potential residual solvent	[120,121]
Nanoprecipitation	PLGA is dissolved in a water-miscible organic solvent and added to an aqueous solution under stirring, leading to nanoparticle formation as the solvent diffuses and precipitates the polymer.	- Simple and rapid - No need for high shear forces	- Limited to hydrophobic drug encapsulation - Potential for broad particle size distribution	[120,121]
Spray Drying	A solution or suspension of PLGA and drug is sprayed into a chamber with heated air, causing solvent evaporation and formation of dry nanoparticles.	- Scalable for large production - Suitable for heat-stable compounds	- High temperatures may degrade sensitive drugs - Potential for broad particle size distribution	[121]
Coacervation	Inducing phase separation of PLGA from a solution by adding a non-solvent, leading to nanoparticle formation.	- High encapsulation efficiency - Solvent-free process	- Difficult control over particle size - Process complexity	[121]
Microfluidics	Utilizing microfluidic devices to mix PLGA and drug solutions under controlled conditions, allowing precise nanoparticle formation.	- Precise control over particle size - High reproducibility	- Requires specialized equipment - Potentially high costs	[119]

Chitosan (CS). In PEG-PLGA systems, chitosan is used primarily as a surface coating, allowing the stealth effect of PEG to be combined with a biologically active surface capable of interacting with bacterial membranes. Several studies have shown that these systems have a much higher antibacterial activity than non-chitosan-coated PLGA nanoparticles. In research by Arafa M.G. et al., ciprofloxacin-loaded PLGA-CS nanoparticles were created to treat root canal infections, noting a large increase in antibacterial efficacy against oral pathogens [122]. These results are due to the combination of controlled drug release and the electrostatic interaction of chitosan with the bacterial wall. It was also seen in a study by Azzazy H.M.E. et al., that PLGA-CS systems loaded with natural alkaloids show strong antibacterial activity with improved wound healing processes [123]. These results suggest that chitosan not only decreases microbial load but also plays an important role in modulating the local inflammatory response.

ε-poly-L-lysine (ε-PL). ε-PLL is a type of natural cationic polymer with antimicrobial action, highly valued for its broad-spectrum effect on bacteria and its biocompatibility. Lee D. U. et al. studied the antimicrobial effect of ε-PLL, highlighting a strong electrostatic interaction with the surfaces of negatively charged bacteria, leading to membrane destabilization and cell death [124]. Although the study does not properly focus on nanoparticle compositions, the chemical and physical characteristics of ε-PLL, such as the fact that it is a polycation and dissolves in water, make it a good element to insert into polymeric nanocarriers such as PEGylated nanoparticle systems or polymeric micelles. Therefore, ε-PLL can potentially be inserted into micellar structures to enhance antibacterial action without losing its biocompatibility.

Polyethylenimine (PEI). PEI is a positively charged polymer, studied for its antimicrobial properties, mainly due to the strong electrostatic reaction with bacterial membranes. Zou Y. et al. examined the design and applications of PEI-based nanogels, showing how structural modification, molecular weight control, and PEGylation methods can reduce cytotoxicity while maintaining high efficacy against bacteria [125]. Although the study does not focus specifically on PEG-PLGA nanoparticles, it demonstrates that PEI can be effectively enclosed within polymeric nanostructures to have controlled action and improved biocompatibility.

Zwitterionic polymers. In the study by Dasanayake G.S. et al., surface-modified PEG-PLGA nanoparticles are created with an imidazolium-based zwitterionic ionic liquid [126]. The zwitterionic modification gives more stability to the particles and reduces the interaction with plasma proteins, maintaining the ability to encapsulate and release the drug. The data show that functionalizing PEG-PLGA with zwitterions is a useful way to overcome the problems of regular PEG, improving the antifouling performance and safety of polymer drug delivery carriers.

### 3.3. Polymeric Micelles

Polymeric micelles are amphiphilic micelles which self-aggregate into nanocarriers while consisting of both hydrophobic and hydrophilic organic structures [127]. Thus, in an aqueous environment, the hydrophobic elements combine into a core with the hydrophilic elements forming a microsphere to create a shell that is very hard to break (Table 6). Polymeric micelles have been hypothesized to be one of the highly promising nanostructures in the delivery of the antimicrobial medicines because of their superior bioavailability, targeting, and controlled release of therapeutic agents. Such a design enables us to bypass the problems resulting from the low bioavailability or the rapid degradation of most of the antimicrobials, and at the same time, provides possibilities for the reduction of antimicrobial resistance development. New engineering has resulted in multifunctional micelles that respond to stimuli such as pH and bacterial enzyme variables [128], hence, facilitating greater control over the antimicrobial payload at the body’s infection sites. These newer generations of micelles are designed for effective target delivery in infected biological microenvironments and improve drug bioavailability while preventing resistance to antibiotics. In addition to improving the bioavailability and stability of the active ingredients, polymer micelles play an essential role in enhancing the drug’s effectiveness against resistant pathogens. In a study by Morteza M. et al., micelles based on PEG-PLGA loaded with piperacillin/tazobactman show a reduction of the MIC against multidrug-resistant Pseudomonas aeruginosa [129].

Polymeric micelles can act through different mechanisms of action. In particular, a recent study by Stancheva R. et al. used mixed polymeric micelles loaded with ciprofloxacin, highlighting a double synergistic mechanism of action [130]. The micellar system, on the one hand, improved the solubility and bioavailability of the antibiotic, and on the other hand, an intrinsic antibacterial activity of the cationic crowns of the mycelium was highlighted. This facilitated electrostatic interaction with the membrane of Gram-negative bacteria, resulting in inhibition of growth.

**Table 6:** Methods to prepare polymeric micelle-based formulations.

Method	Description	Advantages	Disadvantages	Ref.
Dialysis	The amphiphilic polymer is dissolved in an organic solvent miscible with water. This solution is then dialyzed against water or another selected solvent, leading to the gradual removal of the organic solvent and the self-assembly of micelles.	- Gentle method suitable for sensitive molecules. - Allows good control over micelle size. - Does not require surfactants.	- Relatively slow process. - Requires large volumes of solvent for dialysis. - May not be suitable for large-scale production.	[131]
Solvent Evaporation	The polymer is dissolved in a volatile organic solvent immiscible with water. This solution is then added to water under agitation to form an oil-in-water emulsion. The organic solvent is subsequently evaporated, leading to micelle formation	- Relatively simple process. - Suitable for loading hydrophobic drugs. - Ability to control micelle size by adjusting emulsification conditions.	- Use of potentially toxic organic solvents. - Possible residual solvent in the final product. - Requires efficient solvent removal to ensure product purity.	[132]
Direct Dissolution	The amphiphilic polymer is directly dissolved in water or an aqueous solution. The hydrophobic segments of the polymer spontaneously aggregate to form the micelle core, while the hydrophilic segments form the outer corona.	- Simple and rapid method. - Does not require organic solvents. - Suitable for polymers with high water solubility.	- Limited to water-soluble polymers. - Potential formation of unwanted aggregates if polymer concentration is not properly controlled. - Less control over micelle size and morphology compared to other methods.	[133]
Co-solvent Methods	The polymer is dissolved in a mixture of organic solvents and water. The organic solvent is then removed by evaporation or dialysis, inducing the self-assembly of micelles in the remaining aqueous phase.	- Allows control over micelle size by varying solvent composition. - Suitable for a wide range of polymers, including those less soluble in water. - Capability to incorporate various types of drugs.	- Use of potentially toxic organic solvents. - Requires careful solvent removal to avoid residues in the final product. - More complex process compared to direct dissolution.	[133]

Chitosan. The conjugation of hydrophobic chains to the chitosan structure allows the spontaneous formation of stable micelles in the presence of water. Kumar R. et al. derived polymeric micelles from oleic acid-modified carboxymethyl-chitosan [134]. The results showed that the obtained micelles had an average size of 213.4 ± 9.6 nm and a docetaxel encapsulation efficiency of 57.26 ± 1.25%. Furthermore, in vitro release studies have demonstrated controlled and sustained drug release. In parallel, in vivo pharmacokinetic studies showed a 1.97-fold increase in C_max_ and a 2.62-fold increase in AUC, confirming a marked improvement in the oral bioavailability of the micellar formulation compared to free docetaxel.

Similarly, in a study by Chen T. et al., complex micelles were created by combining chitosan with stearic acid; the resulting system could deliver highly hydrophobic drugs [135]. Although primarily developed for drug delivery, these micellar systems provide a versatile platform that can be adapted for antimicrobial applications.

ε-poly-L-lysine (ε-PL). In a study by Yu H et al., amphiphilic copolymers of ε-PL modified with succinic anhydride (M-EPL) were developed [136]. These are capable of self-assembling into spherical polymer micelles in water with diameters of approximately 2.4–2.6 nm. These micelles can encapsulate hydrophobic elements by increasing the solubility and stability of these elements. Furthermore, they can maintain antimicrobial activity specific to ε-PL. This evidence suggests that the micellar system can function both as a nanocarrier and as an antimicrobial agent.

Zwitterionic polymers. The addition of zwitterionic segments to the micellar corona helps to greatly decrease non-specific protein uptake and bacterial adhesion, curbing biofilm development and increasing the colloidal stability of the system. In a study by Tian S. et al., pH-sensitive zwitterionic shell polymer micelles were created, produced by the spontaneous union of zwitterionic PCL-based and PEG-PCL copolymers, capable of charge switching in an acidic environment typical of biofilms [137]. This process improved biofilm penetration against *Staphylococcus* spp., disruption of the extracellular matrix, and a strong increase in the efficacy of ciprofloxacin both in vitro and in vivo. In another study, polymer micelles functionalized with zwitterionic parts and hydrophobic antimicrobial sections were created, to react to bacterial enzymes, allowing the release of antimicrobial agents within the biofilm and causing a significant decrease in bacterial viability in resistant strains [138].

Polyguanidine. The polyguanidine segments are in the nucleus of the micelle or on the surface, offering an interaction with the bacterial membrane. As demonstrated by Ikeda T. et al., polymers with biguanidine sections show MIC values between 2 and 16 μg/mL against Gram-positive bacteria, while incorporation into micellar systems allows for antibacterial efficacy by decreasing damage to eukaryotic cells [139]. Other research has shown that polyguanidine micelles lead to loss of membrane potential and cytoplasmic leakage, with less than 1% viable bacteria after 2 h of exposure [140].

### 3.4. Dendrimers

Dendrimers are branched polymer macromolecules which have a symmetrical three-dimensional architecture containing a central core and outwardly protruding branches (Table 7). This structure facilitates a plethora of functional groups on the exterior, which can be chemically altered in order to activate or boost certain biological activities. Such dendrimers, which include polyamidoamine (PAMAM) [140], polyglycerol dendrimers and PEG-based dendrimers are garnering attention in the medicine field, more so in treating microbial infections. Their high tendency to self-assemble and micronized surface give them excellent for encapsulating and delivering drug-targeting systems. Regarding the antimicrobial applications, dendrimers have some notable benefits. Dendrimers have an advantage in that they can encapsulate hydrophobic antimicrobials within their hydrophobic core [141], thus shielding them from degradation and perhaps increasing their retention. Also, polymethacrylates can have cationic groups, like quaternary amines, conjugated on their surface, which has an antimicrobial effect by itself.

These positively charged groups interact with bacterial membranes, creating disruptions in their structure and promoting cell lysis, a strategy that makes them particularly effective against both Gram-positive and Gram-negative bacteria, including those resistant to traditional antibiotics [140]. In a recent study, Holmes A.M. et al. compared several generations of PAMAM dendrimers (G2-G5) against opportunistic pathogens, including *S. aureus* and *P. aeruginosa*, demonstrating generation-dependent action [142]. Antimicrobial activity increases with dendrimer generation. In particular, the dendrimer G5-PAMAM-NH showed a MIC of 2.9 μg/mL against *S. aureus*, compared to 26.8 μg/mL for G2. The electrostatic interaction between the cationic ends of the dendrimers and the bacterial surface contributed to the bactericidal action, causing permeability and cell death [130,142].

**Table 7:** Methods to prepare dendrimers.

Method	Description	Advantages	Disadvantages	Ref.
Divergent synthesis	Starting from a central core, the polymer is constructed by adding layers of monomers directed towards the outside.	Construction of large-scale dendrimers	- Difficulty in controlling the final structure - Possible formation of unwanted products	[143,144]
Convergent synthesis	It starts in the periphery. Fragments will be united into a central core.	Reduction of collateral products	- Limited size of the dendrimer - Complex and lengthy summary procedure	[143,144]

Chitosan. In a study by Patrulea V. et al., a method for conjugating antimicrobial peptide dendrimers (AMPDs) to chitosan derivatives via a thiol-maleimide reaction was developed [145]. In this way, systems with antibacterial action against *P. aeruginosa* were created, eliminating cellular toxicity. Subsequently, electron microscopy tests were conducted which showed that these compounds are able to damage both the external and internal barriers of Gram-negative bacteria, suggesting a synergistic mechanism of action between the dendrimer and chitosan [146]. Another innovative method involves the creation of conjugates between PAMAM dendrimers and zwitterionic polymers, such as zwitterionic chitosan (ZWC). This system can function as a pH-sensitive coating for dendrimers [147]. Under physiological conditions, ZWC creates a stable complex with PAMAM dendrimers via electrostatic forces, reducing the cytotoxicity of terminal amines and increasing biocompatibility [148].

ε-Poly-L-lysine (ε-PLL). The use of ε-PLL allows an increase in the antimicrobial activity of dendrimers. In a study by Chen S. et al., it was demonstrated that the integration of ε-PLL into dendrimer systems or dendrimer-based hydrogels could enable the sustained release of ε-PLL and enhance antibacterial activity [149]. Furthermore, this system may contribute to the modulation of the local inflammatory response.

Polyethylenimine (PEI). In a study by Gibney KA. et al., it was described how dendrimers with amino surfaces lead to electrostatic interactions with the bacterial wall and destabilization of membranes. These studies suggest that the presence of polycationic chains may improve the antibacterial efficacy of the dendrimer [80]. Yudovin-Farber et al. described PEI-modified dendrimers with quaternary functional groups or cationic shells with increased antibacterial activity compared to the starting molecule, both against Gram-positive and Gram-negative strains [150].

Zwitterionic polymers. Strategies combining dendrimers and zwitterionic polymers seek to limit biological fouling and cytotoxicity while maintaining interaction with microbial surfaces. A recent study illustrates the characteristics and uses of zwitterionic polymers. Moayedi S. et al., clarify how these materials, including carboxybetaines, phosphorylcholine, and sulfobetaines, produce very good surface hydration and prevent protein and bacterial uptake [33]. Dendrimer joining is thought of as core–shell systems where a cationic dendrimer is covered by zwitterionic chains to modulate surface charge in physiological situations.

### 3.5. Medical Composites

Antimicrobial polymers or polymer composites have witnessed considerable interest in the field of biomedical engineering due to their capacity to consolidate the mechanical properties of the polymers with the antimicrobial effect of the agents that have been incorporated within them. Over the past few years, progress in the field of medical composites has concentrated on the development of antimicrobial polymer composites without any loss of the desired mechanical properties. Composites have been developed whereby matrix polymers, such as polylactic acid (PLA), are used in conjunction with antimicrobial nanoparticles like silver or zinc to improve the strength and durability of medical devices [151]. More recently, attempts have been made to use bioresorbable polymers such as poly(ε-caprolactone) (PCL) incorporated with natural antimicrobial peptides for construction of medical devices which would prevent infection and at the same time biodegrade after use, avoiding pollution and enhancing the safe use of the materials in the long term. In addition, biocomposites made of natural polymer matrix and antimicrobial agents can suppress biofilm growth and bacterial growth on different surfaces. These biocompatible composite materials can be used as substitutes for traditional synthetic polymers and can be utilized in regenerative medicine and advanced medical devices for instance [152]. There are many mechanisms by which silver nanoparticles (AgNPs) lead to the breakdown of the bacterial membrane. These include the formation of reactive oxygen species (ROS) and direct interaction with nucleic acids. In a recent study by Gunes C. et al., it was shown that PCL/PLA composites containing AgNPs encapsulated with the essential oil of *Thymus vulgaris* possessed significant antibacterial activity against *E. coli* and *S. aureus*, with a reduction in bacterial load, using 50 μg/mL AgNPs, within 8 h of exposure [153].

Another interesting study by Demchenko V. et al. showed how nanocomposites, based on PLA and chitosan enriched with silver nanoparticles, generated inhibition zones of about 25 mm against the same micro-organisms, also demonstrating antiviral activity against influenza strains and HSV-1, without cytotoxic effects on eukaryotic cells [154].

### 3.6. Surfactants and Flocculants

In order to improve the efficacy and functionality of antimicrobial polymers, surfactants and flocculants are used in biomedical applications.

#### 3.6.1. Surfactants

A surfactant is a molecule that can reduce surface tension by acting as a dispersant, wetting agent, emulsifier and detergent, targeting the interfaces between solids, liquids and gases. The presence of surfactants is essential when dealing with interactions of apparently incompatible phases. Therefore, an important advantage is their ability to maintain a high stability of dispersed phases, this allows the regulation of energy exchange in both synthetic and natural processes. A dispersed system is characterized by the presence of a dispersed phase divided into discrete units within a continuous phase. As described by Cortes H. et al., in the production of nanoparticles reserved for biomedical use, surfactants ensure a stable liquid/liquid system [155]. This system makes the transition to a colloidal solid/liquid system easier.

Surfactants have become an essential means of ensuring stability in aqueous environments and are used for this purpose at all stages of the production process of nanoparticles.

Recent studies have shown that polyhexamethylenediguanide (PHMB) is able to exploit the electrostatic interactions with the negatively charged membranes of bacteria and fungi. This leads to disorganization of the phospholipid bilayer, increased permeability and subsequent cell lysis. In a study by Chindera K. et al., it was shown that PHMB is able to penetrate intact bacterial cells with nuclear penetration, leading to chromosome DNA condensation [156]. This activity was particularly effective against *E. coli*, with MIC values on the order of 1–5 μg/mL, and against other Gram-negative bacteria. PHMB is widely used for topical applications, antimicrobial coating and surface disinfection.

#### 3.6.2. Flocculants

The use of flocculants is to stimulate the aggregation of suspended particles into solutions [144]. This facilitates the separation of these particles through processes such as filtration and sedimentation. In antimicrobial polymer formulations, flocculants can increase the efficiency of pathogenic organisms’ treatment: they lead to an increase in the physical and chemical properties of materials. One of the most widely used flocculants with antimicrobial polymers is cationic polyacrylamide chloride (CPAM) [157]. CPAM is a high-density cationic polymer able to interact with the negative surface charges of micro-organisms, colloids and other suspended particles. In a study by Fu C. et al., single-factor tests were performed to explore the optimal range of factors influencing CPAM flocculation at an early stage [157]. Results show that the flocculation effect was better when the intrinsic viscosity was higher or the cationic degree of CPAM was higher.

A study by Piras et al. suggests that quaternized derivatives of chitosan show high efficacy against both Gram-positive and Gram-negative bacteria [158]. Through chemical modifications to the quaternization of chitosan amino groups, it is possible to increase positive charges and, consequently, the ability to interact with negatively charged bacterial membranes. This interaction leads not only to cell collapse, but also to inhibition of microbial adhesion. Microbiological assays showed MICs between 50 and 100 μg/mL against clinically relevant strains such as *S. epidermidis* and *P. aeruginosa*. It is important to note that an effective anti-adhesive action has also been demonstrated on titanium surfaces, suggesting possible medical applications in the prevention of infections.

### 3.7. Biosensor Coatings

The current research scenario sees biosensor coatings as an increasingly expanding area in immediate application in the biomedical field [159]. These coatings can combine biological recognition capability with the antimicrobial action of polymers. This provides dual protection against microbial contamination, increasing the stability and durability of the biosensor. This is an example of an antimicrobial polymer, already covered during the review, which can inhibit or slow down bacterial proliferation on the biosensor surface is chitosan.

Chitosan. Much research has shown that chitosan-based coatings can block infection related to implantable devices by decreasing bacterial adhesion and biofilm formation on prostheses. In a review by Teixeira-Santos R. et al., it was noted that a chitosan-based coating improves antimicrobial efficacy with important results for implantable medical devices (IMDs) [160]. The obtained materials showed a decrease in bacterial adhesion of up to 70–95% and an inhibition of Staphylococcus aureus and Escherichia coli biofilms, maintaining good cytocompatibility. Regarding wound therapy, Vaz et al. designed porous chitosan-based membranes loaded with polyhexanide (PHMB) by solvent casting and then sterilizing, for use as antimicrobial wound dressings. The membranes showed broad-spectrum antibacterial action and controlled release [161]. Furthermore, in delivery systems for topical uses, chitosan inserted into liposomes or chitosomes increased the antimicrobial action and reduced the inflammatory response, allowing a controlled release of antimicrobial agents such as chlorhexidine. Incorporation of the drug into the polymer resulted in an increase in antimicrobial activity compared to free chlorhexidine, along with a reduction in the local inflammatory response [162].

Zwitterionic polymers. Zwitterionic polymers (such as carboxibetaines, phosphorylcholine, and sulfobetaines) exhibit high hydrophilicity and strong surface hydration that significantly decrease protein adsorption, biofilm creation, and cellular activation on areas of implants and instruments in contact with blood. Moayedi et al. studied the use of zwitterionic polymers as antifouling coatings for medical devices, applied via grafting or surface coating. The modified zones showed a 90% higher reduction in protein adsorption, a sharp drop in bacterial adhesion and reduced macrophage activation [33]. Novel coating strategies use these polymers to modify the surface of device blood-contacting and metal implants, with decreased bacterial and protein adhesion in in vitro tests [163].

PEI and polyguanidines. Regarding cationic polymers such as PEI or guanidine, their application in composites for medical devices exploits the direct electrostatic interaction with microbial membranes. Yue et al. designed composite sponges based on chitosan, PVA, and polyguanidines (PHMG) for wound care applications, achieved by physical cross-linking. The composite materials showed effective bactericidal action against Gram-positive and Gram-negative bacteria, greatly speeding up wound healing in in vivo models and decreasing local inflammation. These findings support the use of cationic polymers in antimicrobial medical devices [164]. Similarly, antibacterial polymer composites and biodegradable polymer structures have enabled the creation of antibiofilm biocomposites for resorbable devices, where the polymer surface is optimized to limit microbial adhesion during the critical post-implantation period [165].

## 4. Conclusions

Antimicrobial polymer formulations are versatile materials with significant potential for infection control. Their main advantage lies in the combination of intrinsic antimicrobial activity and tunable physicochemical properties, which provide different mechanisms of action, including membrane disruption and surface-mediated anti-adhesive effects. Compared with conventional antibiotics, antimicrobial polymers offer broader structural diversity, improved chemical stability, and adaptable functional properties. These features make them suitable for applications such as biomedical coatings, wound care, and drug delivery systems. Their antimicrobial performance depends strongly on polymer composition, molecular architecture, and environmental conditions. In particular, complex biological environments such as biofilms and polymicrobial infections can significantly reduce their efficacy. Antimicrobial polymers represent a promising class of materials for the development of alternative and complementary strategies for infection control.

## 5. Future Perspectives

Despite recent progress, several challenges still limit the clinical translation and large-scale application of antimicrobial polymers. A key requirement is the standardization of material characterization, especially for natural polymers such as chitosan. Important parameters, including molecular weight, degree of deacetylation, charge density, and batch-to-batch variability, should be systematically defined and reported to improve reproducibility.

Further research should focus on the rational design of antimicrobial polymers with improved selectivity and biological performance. Future developments should aim to enhance activity against biofilm-associated bacteria and multidrug-resistant strains while reducing potential toxicity toward host tissues.

Although antimicrobial polymers are generally considered to have a lower risk of inducing classical antibiotic resistance, adaptive bacterial responses such as membrane modification and increased biofilm formation may still occur. These aspects require further long-term investigation.

Another important limitation is the lack of standardized biofilm models. More clinically relevant in vitro systems, including mature and polymicrobial biofilms, are needed to improve the predictive value of experimental results.

From a translational point of view, regulatory classification remains a challenge due to the dual nature of these materials as both biomaterials and active antimicrobial systems. Early consideration of regulatory requirements may facilitate future clinical development.

Finally, scalability and cost-effectiveness are essential for industrial application. Reproducible manufacturing processes, stability during sterilization, and economically viable production routes will be critical for successful translation.

## Data Availability

No new data were created or analyzed in this study. Data sharing is not applicable to this article.
